# Betaine Promotes Milk Protein Synthesis via Alleviating Oxidative Stress Caused by NEFA in Mammary Epithelial Cells of Dairy Cows

**DOI:** 10.1002/vms3.71065

**Published:** 2026-06-28

**Authors:** Wenyan Yan, Xinyuan Sun, Huanjie Shi, Zhenwang Li, Wenhui Li, Xiaoxiao Gao, Jihong Dong

**Affiliations:** ^1^ Laboratory of Animal Nutrition, Metabolic and Poisoning Diseases College of Veterinary Medicine Qingdao Agricultural University Qingdao Shandong China; ^2^ College of Animal Science and Technology Qingdao Agricultural University Qingdao Shandong China

**Keywords:** betaine, bovine mammary epithelial cells, milk protein, N‐acetyl‐L‐cysteine, oxidative stress

## Abstract

**Background:**

Fatty liver is highly prevalent in dairy cows and poses a significant challenge to the dairy industry by reducing both milk yield and quality, thereby inflicting substantial economic losses. Although dietary betaine supplementation has been demonstrated to enhance milk protein content, the underlying molecular mechanisms remain to be fully elucidated.

**Objectives:**

This study aimed to elucidate the role of betaine in milk protein synthesis and the underlying mechanisms.

**Methods:**

Blood, liver and mammary gland samples were obtained from healthy and fatty liver‐affected bovines. The serum NEFA levels, oxidation and antioxidant enzyme systems and milk protein synthesis‐related proteins expression were determined. In vitro, the bovine mammary epithelial cells (BMECs) were pretreated with 25 mM betaine/10 mM N‐acetyl‐L‐cysteine (NAC) and then stimulated with NEFA.

**Results:**

The results indicated an elevation in serum NEFA levels accompanied by enhanced oxidative stress (OS) and reduced protein levels related to milk protein synthesis (phosphorylated (p)‐mechanistic target of rapamycin (mTOR)/mTOR, p‐ribosomal protein S6 kinase 1 (S6K1)/S6K1, p‐Janus kinase 2 (JAK2)/JAK2, p‐signal transducer and activator of transcription 5 (STAT5)/STAT5 and β‐casein) in dairy cows with fatty liver. In vitro, the results revealed betaine decreased the hydrogen peroxide (H_2_O_2_), malondialdehyde (MDA), and oxygen free radicals (OFR) contents, and lactate dehydrogenase (LDH) activity in the supernatant, but increased the glutathione peroxidase (GPX), catalase (CAT), superoxide dismutase (SOD) and thioredoxin reductase (TrxR) activities; the reduced glutathione‐to‐oxidised glutathione (GSH/GSSG) ratio; and the total antioxidant capacity (T‐AOC); and elevated protein levels related to milk protein synthesis in NEFA‐treated BMECs.

**Conclusions:**

These findings indicate that betaine reduces NEFA‐induced OS and enhances milk protein synthesis, suggesting its potential as a nutritional intervention for enhancing milk protein content in dairy cows with metabolic disorders.

## Graphical abstract

Betaine alleviated NEFA‐induced oxidative stress, which served as a central mechanistic link between fatty liver and mammary dysfunction, and concurrently improved milk protein synthesis via regulation of the Nrf2‐JAK2/STAT5/mTOR axis.

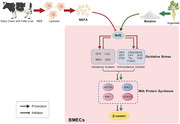

## 1 Introduction

Due to the negative energy balance (NEB), elevated concentrations of non‐esterified fatty acid (NEFA) accumulated as triacylglycerides (TAG; > 5% of the normal lipid content), in the liver of fatty liver cows during the transition period (Herdt 1988). Cows suffering from fatty liver may present a substantial decrease in milk production and other secondary perinatal diseases, which seriously affect fertility and productivity, leading to economic losses and impeding dairy industry development (Glascock and Welch 1974). The large amount of NEFA produced by fat breakdown in cows with fatty liver is lipotoxic, which might be the root cause of oxidative stress (OS), lipid peroxidation, the release of inflammatory factors and mitochondrial dysfunction in various organs (C. Li, Huang, et al. 2022). In bovine mammary epithelial cells (BMECs), high NEFA levels have been shown to induce OS and compromise antioxidant capacity, which is accompanied by decreased milk production and milk protein content (C. Li, Huang, et al. 2022; B. Zhang et al. 2020). Nuclear factor E2‐related factor 2 (Nrf2) acts as the central transcriptional orchestrator governing the expression of cytoprotective genes essential for redox homeostasis (Sun et al. 2020). Previous studies have confirmed that OS can be inhibited by antioxidant supplementation through enabling the Nrf2 signalling pathway in BMECs (Cheng et al. 2021; X. Ma et al. 2022). However, it remains unclear whether high concentrations of NEFA affect milk quality through the Nrf2 pathway.

N‐acetyl‐L‐cysteine (NAC), a synthetic cysteine analogue, is a powerful antioxidant and free radical scavenger to protect cell membranes against lipid peroxidation (Kalyanaraman 2022). It functions as a prodrug that continuously supplies cysteine to cells, facilitating the synthesis of glutathione (GSH)‐a major component of the mammalian antioxidant defence system (Aldini et al. 2018). NAC effectively protects human mammary gland cells and BMECs against reactive oxygen species (ROS)‐induced oxidative damage (Bae et al. 2017; Yu et al. 2022). Given these properties, NAC is widely used as a ROS scavenger and OS protectant.

The alkaloid betaine demonstrates antioxidant capacity akin to that of NAC. Its mechanism involves serving as a methyl donor, which upregulates S‐adenosylmethionine and methionine levels to mediate the antioxidant response. It also regulates osmotic pressure to protect cells and to enhance antioxidant enzyme activity to relieve OS (M. Zhang et al. 2016). Furthermore, betaine can stabilise the phospholipid bilayer and decrease the formation of free radicals and lipid peroxidation of cell membranes to reduce cell damage (Arumugam et al. 2021). Previous research has demonstrated that betaine advanced the ability of mouse granulosa cells subjected to hyperglycaemia to counteract OS by regulating Nrf2 transcription levels (Abnosi et al. 2023). Pretreatment of BMECs with betaine alleviated the lipopolysaccharide‐induced decrease in Nrf2 and haem oxygenase 1 (HO‐1) protein expression (Zhao et al. 2022). Therefore, betaine could inhibit the occurrence of OS by regulating the transcriptional level of Nrf2. However, whether betaine could inhibit OS through the Nrf2 pathway and subsequently promote milk protein production in BMECs remains unclear.

This study evaluated OS and milk protein synthesis indicators in bovine mammary tissue affected by fatty liver in vivo, and the influence of betaine on OS and milk protein synthesis in BMECs in vitro. Our findings provide deeper insights into the pathogenesis of fatty liver‐associated mammary dysfunction and suggest betaine supplementation as a viable preventive measure for periparturient cows.

## 2 Materials and Methods

### 2.1 Animal Selection, Grouping and Tissue Sampling

Experimental animals were selected based on an established protocol from prior research (Dong et al. 2022). Briefly, 120 lactating dairy cows were sourced from a local dairy farm. Blood samples without anticoagulants were collected by jugular venous puncture for 3 days before morning feeding, which were centrifuged and stored at −80°C. Bovine liver tissue (0.2 g) was collected and homogenised with 1 mL of lysis buffer and stored at −80°C. Routine physical examinations for all cows were made to guarantee absence of other comorbidities. All cows were freely fed the same diet (Table S1). According to glucose (GLU), β‐hydroxybutyrate acid (BHB) and NEFA serum levels, as well as liver TAG content, 10 healthy and 10 fatty liver‐affected cattle were selected for this experiment. The baseline characteristics of control cows and dairy cows with fatty liver are presented in Table S2. Right mammary tissue (1–2 g) was collected from each cow, frozen in liquid nitrogen immediately and maintained at −80°C. Three samples were randomly selected for western blotting.

### 2.2 Cell Isolation, Culture and Treatments

Cells were cultured according to the protocol described in a prior study (Yue et al. 2022). Succinctly, BMECs were adjusted to a density of 1 × 10^6^ cells per well in Dulbecco's Modified Eagle Medium (DMEM)/F‐12 (SH30023.01; Hyclone Laboratories, Logan, UT) containing 100 IU/mL penicillin/streptomycin (MA0110; Meilunbio, Dalian, China) and 10% foetal bovine serum (FB15015; Clark, Richmond, USA). DMEM/F‐12 without serum was added for 6 h when BMECs reached 85% of the pore area. Based on serum NEFA concentrations relevant to pathophysiology observed in cows with clinical fatty liver, cells were stimulated with 0, 0.3, 0.6 or 1.2 mM NEFA for 12 h, consistent with previous findings (Bobe et al. 2004; Yue et al. 2022). Subsequently, cells were treated with 25 mM betaine or 10 mM NAC for 2 h or 3 h. The supernatant and pellet were collected and stored at −80°C. In addition, 5 × 10^3^ BMECs were seeded in 24‐well plates containing slides with complete DMEM/F‐12. Cells were processed as described above and subjected to immunofluorescence to observe Nrf2 localisation. There were three replicates per set.

### 2.3 Determination of Blood and Liver Parameters

The GLU, BHB and NEFA serum concentrations were determined using a Hitachi 7170 Automated Analyser (Hitachi) and commercially available kits (GLU: catalogue no. GL3815; BHB: catalogue no. RB1008; NEFA: catalogue no. FA115; Randox Laboratories, Crumlin, UK). The TAG content in the supernatant of liver tissue homogenate was measured with an enzymatic kit (E1013; Applygen Technologies Inc., Beijing, China).

### 2.4 Detection of OS Indicators

The hydrogen peroxide (H_2_O_2_), malondialdehyde (MDA), oxygen free radicals (OFR) and thioredoxin (Trx) contents; the catalase (CAT), glutathione peroxidase (GPX), glutathione S‐transferase (GST), lactate dehydrogenase (LDH), peroxidase (POD), superoxide dismutase (SOD) and thioredoxin reductase (TrxR) activities; the reduced glutathione‐to‐oxidised glutathione (GSH/GSSG) ratio; and the total antioxidant capacity (T‐AOC) were determined with commercially available kits (Shanghai Enzyme‐linked Biotechnology Co. Ltd., Shanghai, China).

### 2.5 Western Blotting

Tissue and BMECs were lysed in radioimmunoprecipitation assay buffer (Applygen, Beijing, China). After quantifying protein concentrations using a BCA assay kit (Applygen, China), proteins were resolved via sodium dodecyl sulphate–polyacrylamide gel electrophoresis. Following electrotransfer to a polyvinylidene difluoride membrane (0.45 µm, Millipore, Bedford, MA), the membrane was incubated in a solution with 5% non‐fat milk powder (Sangon, Shanghai, China) for 2 h. Then, the membrane was incubated with the primary antibody overnight. Primary antibodies: mechanistic target of rapamycin (mTOR) (1:1000, bs‐1992R, Bioss, Beijing, China), phosphorylated (p)‐mTOR (1:1000, AF3308, Affinity Biosciences, Jiangsu, China), Janus kinase 2 (JAK2) (1:1000, bs‐0908R, Bioss), p‐JAK2 (1:1000, YP0155, ImmunoWay, Plano, USA), Nrf2 (1:1000, bs‐1074R, Bioss), signal transducer and activator of transcription 5 (STAT5) (1:1000, bs‐1142R, Bioss), p‐STAT5 (1:1000, bs‐1659R, Bioss), ribosomal protein S6 kinase 1 (S6K1) (1:1000, ab9366, Abcam, Cambridge, UK), p‐S6K1 (1:1000, YP1427, ImmunoWay), β‐casein (1:1000, bs‐10032R, Bioss) and β‐actin (1:5000, P60709, Abways, Shanghai, China). The membrane was incubated with horseradish peroxidase‐conjugated antibody (1:5000, Bioss). After further washing, the membrane was incubated with ECL ultrasensitive luminescence solution (Affinity Biosciences), and images were captured with a protein imager (Protein Simple, San Jose, CA, USA). ImageJ software (National Institutes of Health, Bethesda, MD, USA) was used for densitometric analysis.

### 2.6 Immunofluorescence to Assess Nrf2 Localisation in BMECs

Immunofluorescence was used to analyse Nrf2 localisation in BMECs. BMECs cultured on glass slides were fixed with 4% paraformaldehyde solution and subsequently blocked with goat serum. The slides were incubated overnight at 4°C with Nrf2 antibody (1:300; bs‐1074R, Bioss) in goat serum, followed by fluorescently labelled antibody (1:100; AS053, ABclonal, Wuhan, China) for 1 h at 37°C. After washing, cell nuclei were stained in the absence of light for 10 min with diamidino‐2‐phenylindole. Nrf2 localisation was observed with a laser scanning confocal microscope (Zeiss, Oberkochen, Germany).

### 2.7 Statistical Analysis

Data were analysed using SPSS Statistics 26.0 (IBM, Armonk, NY, USA), with results presented as mean ± SEM. Animal experiment data with a normal distribution were analysed using an independent‐samples *t*‐test, and cell biochemistry and western blotting data were assessed by the one‐way analysis of variance with LSD. The GraphPad Prism 8.0 software (GraphPad Software, San Diego, CA, USA) and BioGDP were used to prepare graphs. Statistical significance was assessed at *p* < 0.05.

## 3 Results

### 3.1 Effects of Fatty Liver on OS and Milk Protein Synthesis in Dairy Cows

Cows in the healthy and fatty liver group were of similar parity, day in milk, body weight and body condition scores (*p* > 0.05, Table S2). However, the NEFA and BHB serum levels, as well as the liver TAG content, were greater; the milk production, dry matter intake and GLU serum levels were reduced in cows with fatty liver (*p* < 0.05, *p* < 0.01, Table S2).

**FIGURE 1:**
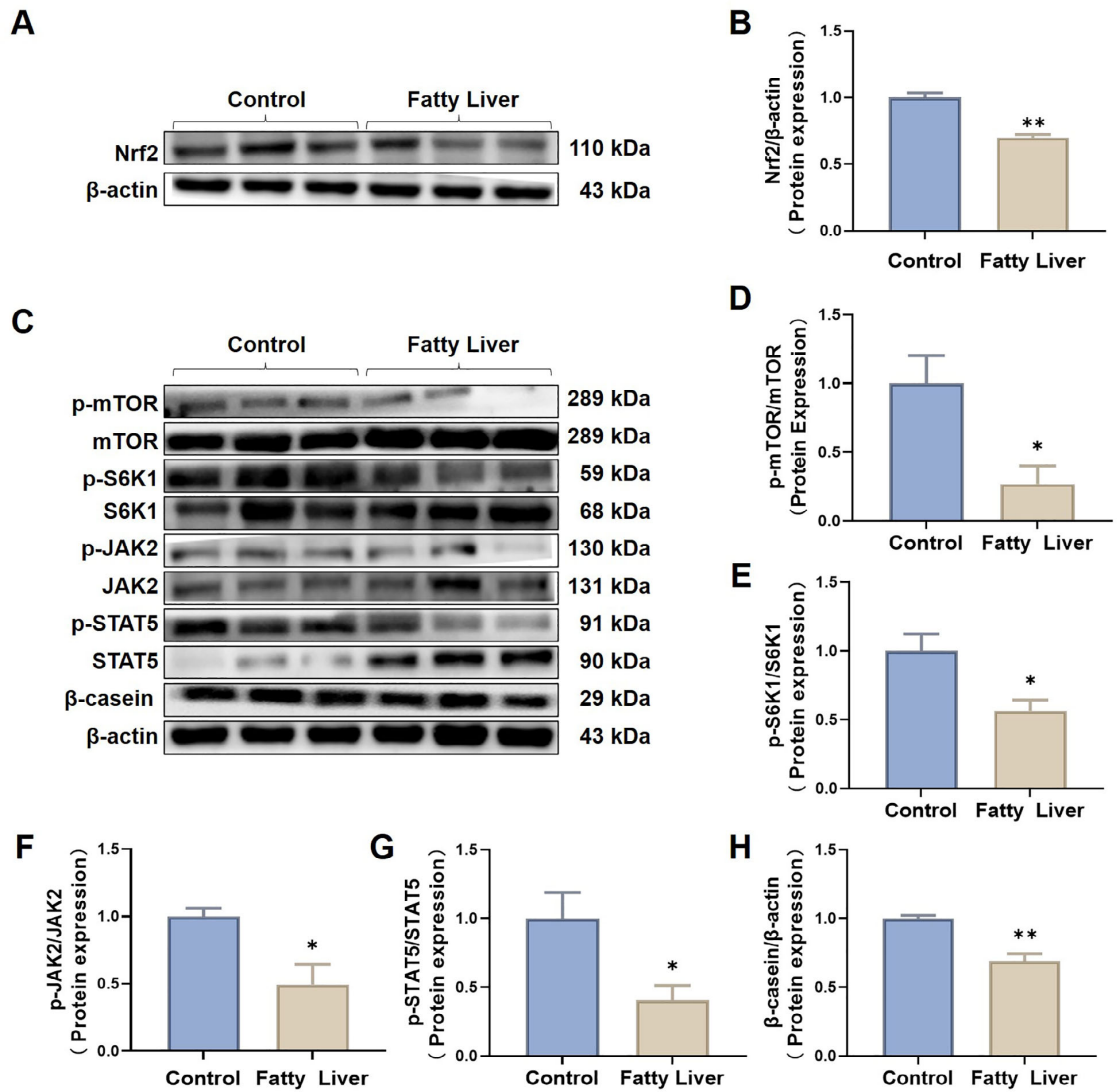
Effects of fatty liver on OS and milk protein synthesis in dairy cows. (A) Western blotting analysis of Nrf2, with β‐actin used for normalisation. (B) Quantification of Nrf2. (C) Western blotting analysis of the expression of proteins associated with milk protein synthesis (p‐mTOR, mTOR, p‐S6K1, S6K1, p‐JAK2, JAK2, p‐STAT5, STAT5 and β‐casein), with β‐actin used for normalisation. (D–H) Quantification of the protein expression levels associated with milk protein synthesis. Data from the treatment group were normalised to the control. Results represent the mean ± SEM from three independent replicates. ^*^ *p* < 0.05, **p < 0.01.

We assessed the effect of fatty liver on the protein level of Nrf2 in mammary glands. As shown in Figure 1A,B, Nrf2 protein level was obviously reduced in dairy with fatty liver compared with control group (*p* < 0.01). The p‐mTOR/mTOR (Figure 1C,D, *p* < 0.05), p‐S6K1/S6K1 (Figure 1C,E, *p* < 0.05), p‐JAK2/JAK2 (Figure 1C,F, *p* < 0.05) and p‐STAT5/STAT5 (Figure 1C,G, *p* < 0.05) ratios and β‐casein protein expression (Figure 1C,H, *p* < 0.01) were significantly lower in mammary tissues from cows with fatty liver.

### 3.2 Effects of NEFA on OS‐Related Indicators in BMECs

**FIGURE 2:**
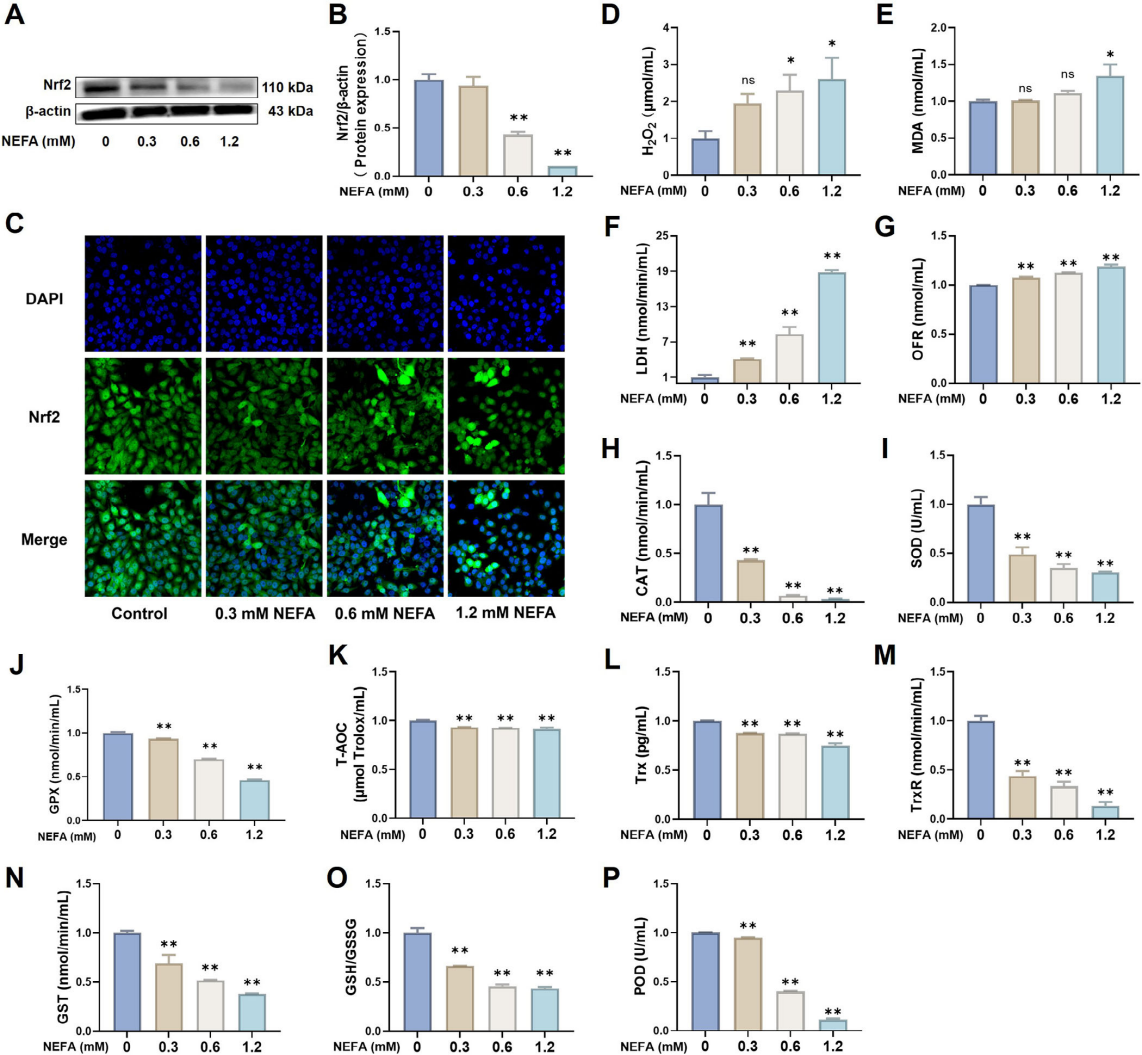
Effects of NEFA on OS‐related indicators in BMECs. (A) Western blotting analysis of Nrf2, with β‐actin used for normalisation. (B) Quantification of Nrf2. (C) Nrf2 immunostaining. (D) H_2_O_2_ content. (E) MDA content. (F) LDH activity. (G) OFR contents. (H) CAT activity. (I) SOD activity. (J) GPX activity. (K) T‐AOC. (L) Trx content. (M) TrxR activity. (N) GST activity. (O) GSH/GSSG ratio. (P) POD activity. The treatment group data were normalised with the control group data. Results represent the mean ± SEM from three independent replicates. ^*^ *p *< 0.05 and ^**^ *p* < 0.01.

We treated BMECs with different NEFA concentrations (0, 0.3, 0.6 and 1.2 mM) for 12 h. In comparison to the control group, Nrf2 protein expression was significantly inhibited in the 0.6 and 1.2 mM NEFA groups (Figure 2A,B, *p* < 0.01), and the amount of Nrf2 entering the nucleus decreased as the NEFA concentration increased (Figure 2C). Relative to the control group, the H_2_O_2_, MDA, OFR contents and the LDH activity were significantly increased (Figure 2D–G, *p* < 0.05, *p* < 0.01) in the 0.3, 0.6 and 1.2 mM NEFA groups. The CAT, SOD, GPX, TrxR, GST and POD activities, the T‐AOC, the Trx content and the GSH/GSSG ratio were substantially decreased (Figure 2H–P, *p* < 0.01) in the 0.3, 0.6 and 1.2 mM NEFA groups.

However, compared with the 1.2 mM NEFA group, the protein expression level of Nrf2 was significantly upregulated and the amount of Nrf2 entering the nucleus was increased (Figure S1A–C). The H_2_O_2_ and OFR contents, the LDH activity were significantly declined (Figure S1D, F, G, *p* < 0.01), while the CAT, SOD, GPX, GST, and POD activities, and the Trx content were notably enhanced in the NAC + 1.2 mM NEFA group (Figure S1H–J,L,N,P, *p* < 0.01).

### 3.3 Effects of NEFA on Milk Protein Synthesis in BMECs

**FIGURE 3:**
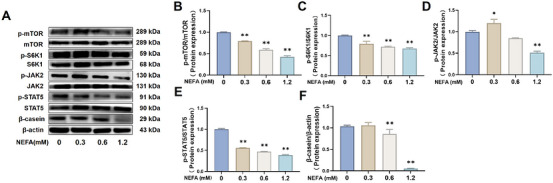
Effects of NEFA on milk protein synthesis in BMECs. (A) Western blotting analysis of the proteins associated with milk protein synthesis, with β‐actin used for normalisation. (B–F) Quantification of the protein expression levels associated with milk protein synthesis. The treatment group data were normalised with the control group data. Results represent the mean ± SEM from three independent replicates. ^*^ *p *< 0.05 and ^**^ *p* < 0.01.

Relative to the control group, the ratios of p‐mTOR/mTOR, p‐S6K1/S6K1, and p‐STAT5/STAT5 (Figure 3A–C,E, *p* < 0.01) were obviously attenuated in the NEFA‐treated groups. The p‐JAK2/JAK2 ratio was considerably enhanced in the 0.3 mM NEFA group (*p* < 0.05), and decreased in the 1.2 mM NEFA group (Figure 3A,D, *p* < 0.01). In addition, β‐casein protein expression (Figure 3A,F, *p* < 0.01) was remarkably inhibited in the 0.6 and 1.2 mM NEFA groups. However, the protein levels of milk protein synthesis in the NAC + 1.2 mM NEFA group were markedly elevated compared with the 1.2 NEFA group (Figure S2A–D,F, *p* < 0.01).

### 3.4 Effects of Betaine on OS‐Related Indicators in BMECs

**FIGURE 4:**
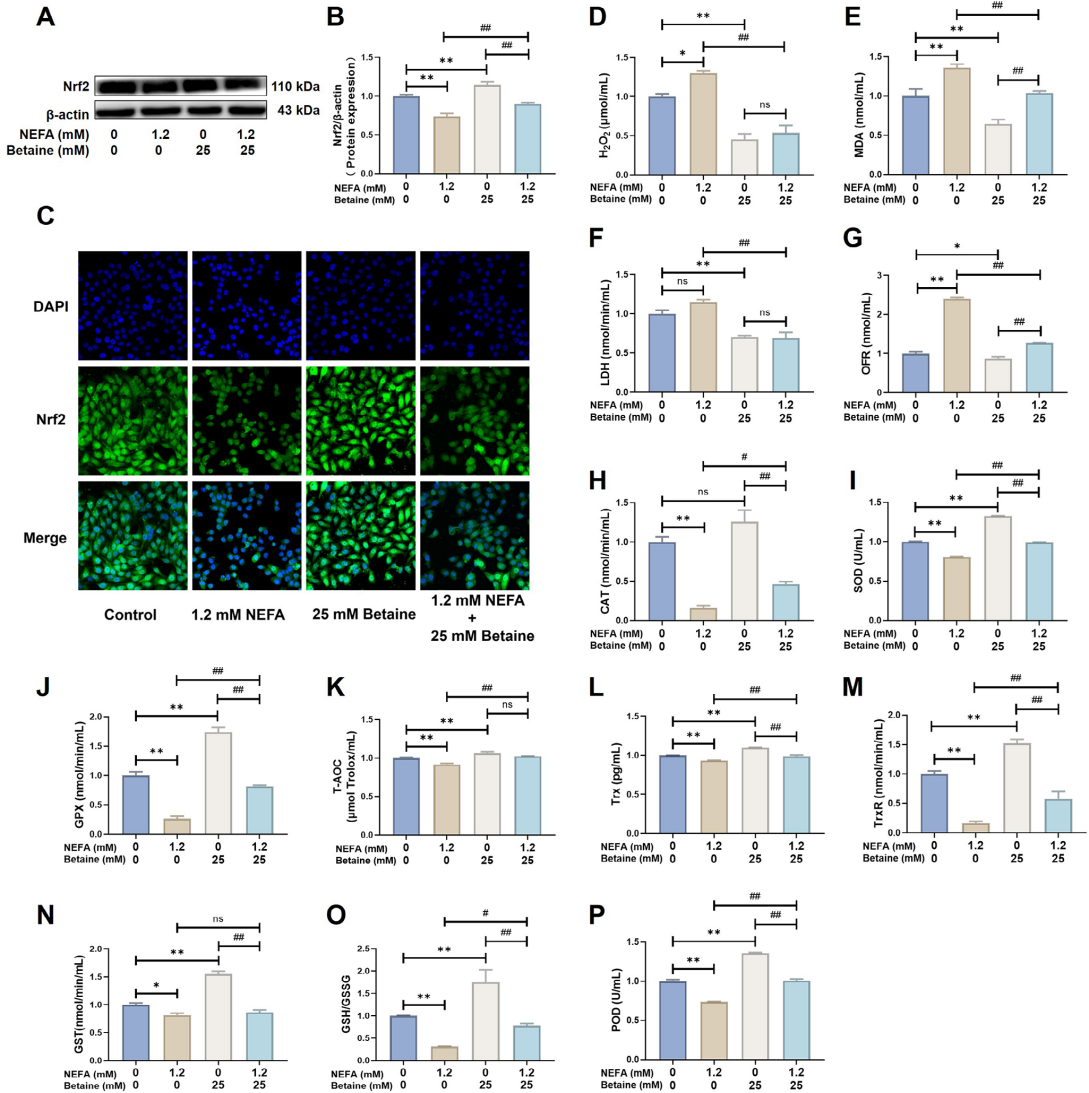
Effects of betaine on OS‐related indicators in BMECs. (A) Western blotting analysis of Nrf2, with β‐actin used for normalisation. (B) Quantification of Nrf2. (C) Nrf2 immunostaining. (D) H_2_O_2_ content. (E) MDA content. (F) LDH activity. (G) OFR contents. (H) CAT activity. (I) SOD activity. (J) GPX activity. (K) T‐AOC. (L) Trx content. (M) TrxR activity. (N) GST activity. (O) GSH/GSSG ratio. (P) POD activity. The treatment group data were normalised with the control group data. Results represent the mean ± SEM from three independent replicates. ^*^ *p* < 0.05, ^**^ *p* < 0.01, ^#^ *p* < 0.05 and ^##^ *p* < 0.01.

To evaluate the influence of betaine on both the expression of Nrf2 and its nuclear accumulation, we pretreated BMECs with 25 mM betaine for 2 h, and then incubated them with 1.2 mM NEFA or without NEFA for 12 h. Compared with the 1.2 mM NEFA group, the abundance of Nrf2 and the amount of Nrf2 in the nucleus were obviously increased in the betaine + 1.2 mM NEFA group (Figure 4A–C, *p* < 0.01). However, Nrf2 protein level and the amount of Nrf2 in the nucleus were markedly reduced in the betaine + 1.2 mM NEFA group compared with the betaine group (Figure 4A–C, *p* < 0.01).

Compared with the 1.2 mM NEFA group, the H_2_O_2_, MDA, OFR contents and the LDH activity, were significantly reduced (Figure 4D–G, *p* < 0.01) in the betaine + 1.2 mM NEFA group. Moreover, the CAT, SOD, GPX, TrxR and POD activities, the Trx content, the GSH/GSSG ratio and the T‐AOC were significantly increased (Figure 4H–P, *p* < 0.05, *p* < 0.01) in the betaine + 1.2 mM NEFA group. Compared with the betaine group, the MDA and OFR contents were significantly increased (Figure 4E,G, *p* < 0.01), while the CAT, SOD, GPX, TrxR, GST and POD activities, the Trx content and the GSH/GSSG ratio were significantly reduced (Figure 4H–P, *p* < 0.01) in the betaine + 1.2 mM NEFA group.

### 3.5 Effects of Betaine on Milk Protein Synthesis in BMECs

**FIGURE 5:**
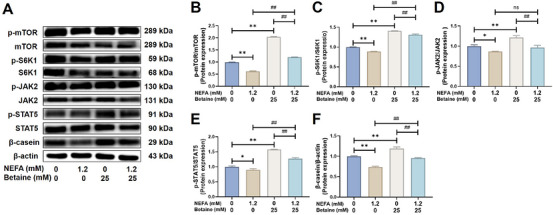
Effects of betaine on milk protein synthesis in BMECs. (A) Western blotting analysis of the proteins associated with milk protein synthesis, with β‐actin used for normalisation. (B–F) Quantification of the protein expressions associated with milk protein synthesis. The treatment group data were normalised with the control group data. Results represent the mean ± SEM from three independent replicates. ^*^ *p* < 0.05, ^**^ *p* < 0.01, ^#^ *p* < 0.05 and ^##^ *p* < 0.01.

Compared with the 1.2 mM NEFA group, the p‐mTOR/mTOR (Figure 5A,B, *p* < 0.01), p‐S6K1/S6K1 (Figure 5A,C, *p* < 0.01) and p‐STAT5/STAT5 (Figure 5A,E, *p* < 0.01) ratios, as well as β‐casein protein expression (Figure 5A,F, *p* < 0.01) were significantly increased in the betaine + 1.2 mM NEFA group. Compared with the betaine group, the protein expressions related to milk protein synthesis were diminished in the betaine + 1.2 mM NEFA group (Figure 5A–F, *p* < 0.01).

## 4 Discussion

Hepatic NEFA accumulation driven by NEB results in the development of fatty liver in dairy cows, which is accompanied by the occurrence of OS (Drackley 1999). The OS inhibits Nrf2 expression, thereby reducing the function of the antioxidant system and milk protein synthesis (C. Li, Huang, et al. 2022; Rojo de la Vega et al. 2016). Therefore, exploring the mechanism of OS caused by NEFA through the Nrf2 pathway could provide valuable information to reverse the altered milk protein synthesis. In this study, we confirmed the following: (1) High NEFA concentrations downregulated Nrf2 protein expression and suppressed the activities of key antioxidant enzymes in the mammary tissues of dairy cows with fatty liver, collectively inducing significant OS in mammary glands. (2) Fatty liver and a high NEFA concentration impaired milk protein synthesis by decreasing the protein expression levels associated with milk protein synthesis in the mammary glands. (3) Betaine alleviated the decreased Nrf2 protein expression and the increased OS induced by a high NEFA concentration, and significantly increased the expression of proteins associated with milk protein synthesis in BMECs.

Nrf2 modulates the transcription of target genes by binding to the antioxidant response element (ARE) to upregulate the expression of Type II detoxification enzymes (antioxidant system enzymes), including SOD, CAT, GSH and the Trx system. The resultant upregulation of these enzymes accelerates the catabolism of superoxide and peroxide and reduces cell damage, as denoted by reduced LDH activity and H_2_O_2_ and OFR contents (Q. Ma 2013). C. Li, Huang, et al. (2022) reported that OS was induced in BMECs by NEFA and BHB, which decreased T‐SOD and GPX activities while increasing the accumulation of ROS and MDA. Yan et al. (2022) demonstrated that NEFA induced OS through a mechanism involving ROS accumulation and subsequent activation of the mitogen‐activated protein kinase (MAPK) pathway. Our results corroborate and extend these earlier observations by showing that high levels of NEFA inhibit Nrf2 protein expression and impair its nuclear translocation in BMECs. These alterations triggered a broad OS response, marked by elevated LDH, H_2_O_2_ and OFR, alongside compromised antioxidant defences as seen in reduced activities of CAT, SOD, GPX, TrxR, GST and POD, decreased levels of Trx, the GSH/GSSG ratio and T‐AOC. In contrast, we found that there were no significant changes in abundance of Nrf2 and the H_2_O_2_ and MDA contents in the low NEFA concentration group. Synthesising our research and previous findings, we believe that low concentrations of NEFA appear to trigger an upregulation of the antioxidant system in BMECs as a compensatory mechanism against OS (Cui et al. 2019; Huang et al. 2021). However, high NEFA concentrations exceed the critical threshold of the antioxidant system, which leads to significant oxidative damage in BMECs.

The mammary epithelium is the primary tissue responsible for lactation. Milk protein synthesis is predominantly regulated by JAK2‐STAT5 and mTOR, with β‐casein constituting the majority of lactoprotein (C. Li, Huang, et al. 2022; F. Li, Hu, et al. 2022). The JAK2‐STAT5 pathway is vital for the transcription of the β‐casein gene. Activation occurs through phosphorylation of JAK2 at Tyr^1007/1008^, which in turn phosphorylates STAT5 at Tyr^694^. Then, STAT5 migrates to the nucleus and associates with the β‐casein promoter at a transcription factor binding site, finally inducing the development of downstream target genes (Glascock and Welch 1974). The mTOR signalling pathway is crucial for β‐casein translation in the mammary glands. mTOR is activated by phosphorylation at Ser^2448^, leading to subsequent phosphorylation of downstream effector S6K1 (Shu et al. 2020; M. C. Zhang et al. 2018). In the mammary tissue of cows with clinical ketosis, the mTOR and JAK2‐STAT5 signalling pathways were inhibited by high levels of fatty acid absorption through decreased p‐JAK2, p‐STAT5, p‐mTOR and β‐casein protein expression (Shu et al. 2020). In addition, elevated ROS induced by heat stress in cows also inhibited the mTOR and JAK2‐STAT5 signalling pathways and decreased milk protein synthesis (Guo et al. 2021). In the present research, we revealed that the mTOR and JAK2‐STAT5 signalling pathways were inhibited in mammary tissue of bovines with fatty liver and in BMECs stimulated with a high NEFA concentration, which caused cell damage and decreased the β‐casein content, ultimately affecting milk protein synthesis. In addition, a low NEFA concentration inhibited the mTOR and JAK2‐STAT5 signalling pathways by decreasing the aforementioned protein ratios. However, this low NEFA concentration did not alter β‐casein protein expression, suggesting that BMECs mount a positive regulatory response to low‐level NEFA exposure without compromising β‐casein content or milk protein synthesis. These results are in line with the alterations observed in OS‐related indicators.

NAC effectively attenuates OS via its well‐established ROS‐scavenging and antioxidant activities in BMECs (Bae et al. 2017). In alignment with this established function, our experimental results demonstrated that NAC administration effectively reduced the levels of H_2_O_2_ and OFR, and LDH activity, caused by NEFA in BMECs, while simultaneously enhancing the activities of key antioxidant enzymes. In addition, the suppression of Nrf2 protein expression and its nuclear translocation was significantly attenuated by NAC administration. The results strongly suggest that NEFA induces OS primarily through suppression of Nrf2 in BMECs. We further investigated the effects of NAC supplementation on milk protein synthesis. As anticipated, NAC had a protective effect on milk protein synthesis by activating the mTOR and JAK2‐STAT5 pathways.

The biological mechanisms of traditional Chinese medicine are closely related to bioactive constituents, including polysaccharides, flavonoids, saponins and alkaloids. These compounds, which can mitigate OS via modulation of pathways including Nrf2, nuclear factor kappa B (NF‐κB) and MAPK/Nrf2/Kelch‐like ECH‐associated protein 1 (Liang et al. 2021; Pasta et al. 2016). Betaine is the primary active ingredient in sugar beets, which can upregulate the levels of Nrf2, CAT, SOD and GPX, and ameliorate high GLU‐induced OS in mouse granulosa cells (Abnosi et al. 2023). Further evidence proves that betaine administration attenuates hepatic and renal oxidative damage by enhancing of GSH, GPX, CAT and SOD activities, coupled with reduction of MDA content in mouse tissues (Pourmehdi et al. 2020). Treatment with 25 mM betaine for 12 h in vitro alleviated OS induced by heat stress by reducing ROS accumulation and increasing SOD and CAT levels in BMECs (C. Li et al. 2019). Similarly, this research found that pretreatment of BMECs with 25 mM betaine for 3 h significantly promoted the nuclear translocation and expression of Nrf2, increased the activity of antioxidant enzymes, and enhanced the antioxidant capacity of cells compared with the control group. Building upon these foundational observations, we showed that betaine could antagonise the decreased antioxidant capacity of BMECs caused by high NEFA concentrations by increasing Nrf2 protein expression. Specifically, the capacities of antioxidant enzymes (CAT, SOD, GPX, Trx, TrxR and POD), the GSH/GSSG ratio, and the T‐AOC in BMECs treated with a high NEFA concentration were obviously enhanced by betaine, while reducing the levels of oxidative damage factors (OFR, H_2_O_2_, MDA and LDH). Previous studies demonstrated that providing dairy cows with a supplement of approximately 15–20 g of betaine daily for 21 days could significantly increase milk production (by approximately 6%) and enhance the absolute output of milk protein in practical production (Dunshea et al. 2019; L. Zhang et al. 2014). A meta‐analysis suggested that betaine supplementation exerts positive effects on milk yield, milk fat yield, milk lactose yield and other production traits in dairy cows (Malik et al. 2024). We verified these findings from an in vitro perspective, showing that betaine pretreatment increased the abundance of proteins related to milk protein synthesis. It further demonstrated that betaine attenuated the inhibition of milk protein synthesis induced by high NEFA concentrations through activating the mTOR and JAK2‑STAT5 signalling pathways and upregulating β‐casein expression. Although NAC exhibited certain antioxidant effects comparable to betaine, comparative analysis confirmed that betaine possesses more potent antioxidant properties, as evidenced by the significantly greater upregulation of Nrf2 expression, nuclear translocation, and β‐casein protein abundance following betaine treatment relative to the control group, whereas the effect of NAC was less pronounced than that of betaine. A critical physiological advantage of betaine for perinatal cows is its safer profile, a benefit owing in part to its traditional use in Chinese medicine. Furthermore, betaine functions as a critical regulator in regulating of osmotic function and methyl metabolism, which is closely related to metabolic challenges such as negative energy balance and OS in postpartum dairy cows. Therefore, we believe that betaine provides a superior protective effect compared with NAC and is more suitable for routine feed supplementation to improve the antioxidant status of perinatal cattle.

The present study revealed that high NEFA levels inhibited milk protein synthesis through oxidative damage, whereas betaine counteracted these effects by activating the protective Nrf2‐mediated mTOR/JAK2‐STAT5 signalling pathway in BMECs. This potentially represents a key mechanism underlying betaine's enhancement of milk protein synthesis. Nevertheless, given the complexity of the physiological environment, metabolic crosstalk, and multiple regulatory networks governing milk protein synthesis in dairy cows, the milk protein experiments of this study may not completely replicate the intricate physiological microenvironment. Further in vivo studies are warranted to verify the actual effects of dietary supplementation with betaine, alone or in combination with other nutritional additives, on OS and milk protein synthesis in dairy cows with fatty liver, thereby providing more specific guidance for subsequent clinical translation. Moreover, the protein interaction mechanism between the antioxidant pathway and the milk protein synthesis pathway can be further explored, thereby providing new ideas and targets for the nutritional regulation and clinical intervention of milk protein synthesis disorders in dairy cows with fatty liver.

## 5 Conclusions

**FIGURE 6:**
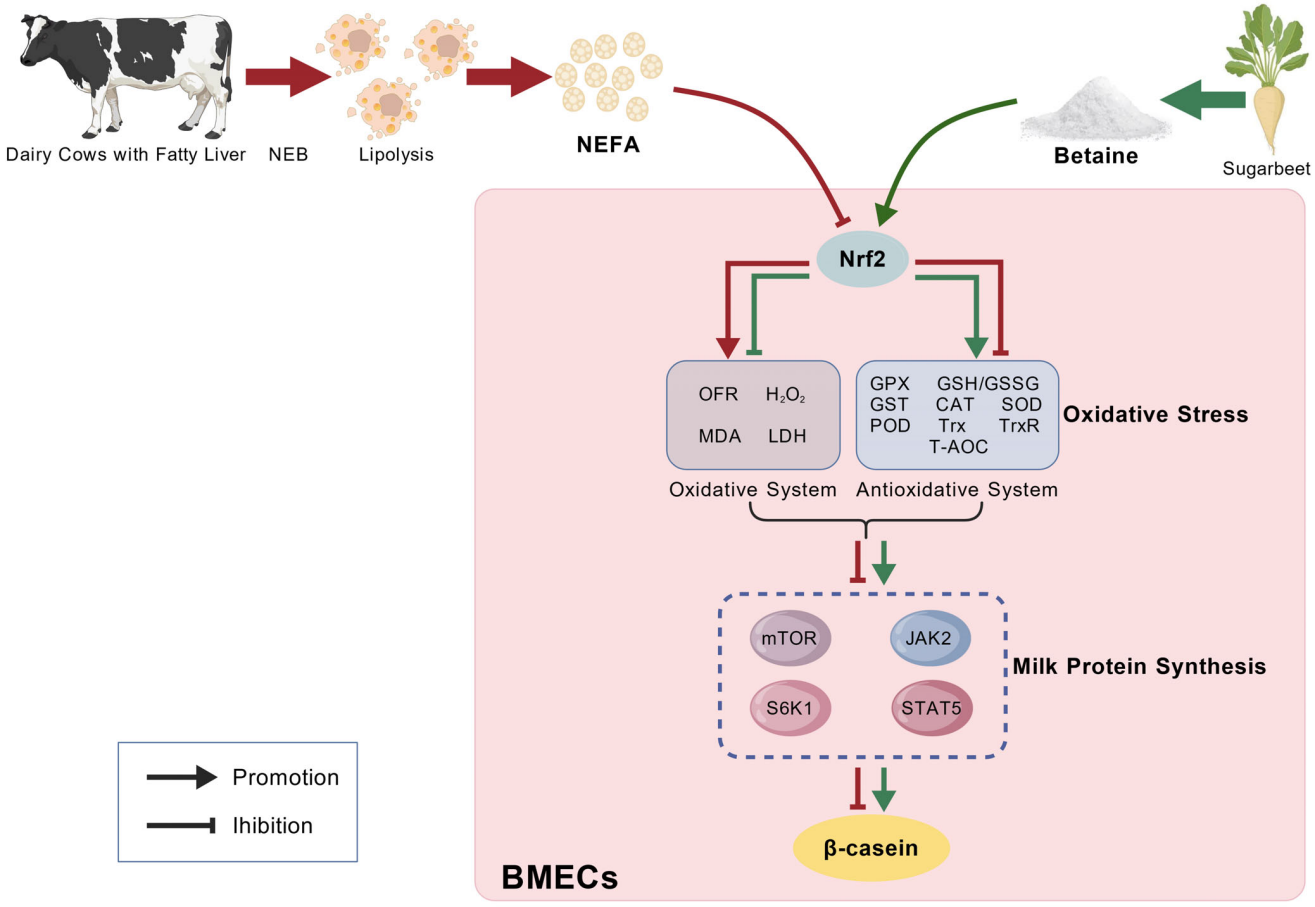
Betaine reduces NEFA‐induced OS and promotes milk protein synthesis through the Nrf2‐mediated mTOR/JAK2‐STAT5 signalling pathway in the mammary tissue of dairy cows with fatty liver.

In summary, betaine alleviated NEFA‐induced OS in BMECs through the Nrf2 signalling pathway and improved milk protein synthesis via regulating the mTOR and JAK2‐STAT5 signal transduction pathways (Figure 6). Our research identifies betaine supplementation as a promising strategy to improve milk protein yield and quality in dairy cows with fatty liver, offering a direct pathway for clinical translation.

## Author Contributions

**Wenyan Yan**: investigation, software, visualisation, writing – original draft. **Xinyuan Sun**: investigation, software, visualisation, writing – original draft. **Huanjie Shi**: formal analysis. **Zhenwang Li**: formal analysis. **Wenhui Li**: formal analysis. **Xiaoxiao Gao**: conceptualisation, methodology, writing – review and editing. **Jihong Dong**: conceptualisation, investigation, software, visualisation, writing – original draft, writing – review and editing, funding acquisition, project administration. All authors read and approved the final manuscript.

## Ethics Statements

The animal study protocol was approved by the Animal Care and Use Committee of Qingdao Agricultural University in accordance with laboratory animal guidelines (GB/T35892‐2018, National Standards of the People's Republic of China). We have obtained informed consent from the owner to use the animals in our study.

## Conflicts of Interest

The authors declare no conflicts of interest.

## Supporting information




**Supplemental Table S1**: The basal diet formulation.
**Supplemental Table S2**: Baseline characteristics of control cows and cows with fatty liver (*n* = 10 per group).
**Supplemental Fig. S1**: Effects of NAC on the oxidation and antioxidant enzyme systems in BMECs. (A) Western blotting analysis of Nrf2, with β‐actin used as a loading control. (B) Quantification of Nrf2. (C) Nrf2 immunostaining. (D) H2O2 content. (E) MDA content. (F) LDH activity. (G) OFR contents. (H) CAT activity. (I) SOD activity. (J) GPX activity. (K) T‐AOC. (L) Trx content. (M) TrxR activity. (N) GST activity. (O) GSH/GSSG ratio. (P) POD activity. The treatment group data were normalised with the control group data. Results represent the mean ± SEM from three independent replicates. *p < 0.05, ** *p* < 0.01, #*p* < 0.05 and ## *p* < 0.01.
**Supplemental Fig. S2**: NAC improved the abundance of milk protein synthesis in BMECs. (A) Western blotting analysis of the proteins associated with milk protein synthesis, with β‐actin used for normalization. (B–F) Quantification of the protein expressions associated with milk protein synthesis. The treatment group data were normalised with the control group data. Results represent the mean ± SEM from three independent replicates. *p < 0.05, **p < 0.01, #*p* < 0.05 and ##*p* < 0.01.

## Data Availability

The data that support the findings of this study are available in the Supporting Information of this article.
