# Supplementary material for: Betaine Promotes Milk Protein Synthesis via Alleviating Oxidative Stress Caused by NEFA in Mammary Epithelial Cells of Dairy Cows
Source: Vet Med Sci. 2026 Jun 28;12(4):e71065. doi: 10.1002/vms3.71065 (PMC13310464; doi:10.1002/vms3.71065)

**Supplemental Table S1.** The basal diet formulation.

| Item (% of DM) | Amount |  |  |
| --- | --- | --- | --- |
| Corn silage | 40.00 | |  |
| Corn | 35.00 | |  |
| Wheat bran | 8.00 | |  |
| Soybean meal | 5.00 | |  |
| Sunflower | 8.00 | |  |
| NaCl | 1.00 | |  |
| Premix* | 1.80 | |  |
| NaHCO_3_ | 1.20 | |  |
| Total | 100.00 | | |
| Nutrient composition (% of DM) |  | | |
| NE_L_(MJ/kg) | 6.70 | | |
| CP | 15.20 | | |
| NDF | 33.45 | | |
| ADF | 17.20 | | |
| NFC | 40.40 | | |
| Ca | 0.70 | | |
| P | 0.50 | | |

^*^One kg of premix contained the following: Vitamin A 200,000 IU, Vitamin D 70,000 IU, Vitamin E 1,000 IU, Fe 2 000 mg, Cu 600 mg, Zn 2,400 mg, Mn 1,300 mg, I 6 mg, Co 7 mg.

DM, Dry Matter; NE_L_, Net energy for lactation; CP, Crude protein; NDF, Neutral detergent

fiber; ADF, Acid detergent fiber; NFC, Non-fiber carbohydrate.

**Supplemental Table S2.** Baseline characteristics of control cows and cows with fatty liver (n=10 per group). Results represent the median ± interquartile range (IQR).

|  | Control cows | | Cows with fatty liver | |  |
| --- | --- | --- | --- | --- | --- |
| Item | Median | IQR^1^ | Median | IQR | P-value |
| Parity | 3 | 2-4 | 3 | 2-4 | p>0.05 |
| DIM^2^, d | 7 | 5-10 | 7 | 5-10 | p>0.05 |
| Body weight, kg | 654 | 624-681 | 675 | 626-694 | p>0.05 |
| BCS^3^ | 3.17 | 3.00-3.21 | 3.23 | 3.13-3.30 | p>0.05 |
| Milk, kg/d | 34.7 | 33.2-37.1 | 30.4 | 28.6-32.3 | p<0.01 |
| DMI^4^, kg/d | 20.5 | 19.3-20.9 | 18.6 | 17.5-20.0 | p<0.05 |
| Glucose, m*M* | 4.06 | 3.99-4.20 | 3.04 | 2.97-3.15 | p<0.01 |
| NEFA, m*M* | 0.31 | 0.26-0.36 | 0.79 | 0.71-1.06 | p<0.01 |
| BHB, m*M* | 0.43 | 0.28-0.51 | 1.69 | 1.29-1.85 | p<0.01 |
| TAG^5^, % liver wt | 0.57 | 0.36-0.64 | 5.57 | 3.09-6.91 | p<0.01 |

^1^IQR: Interquartile range. ^2^DIM: day in milk. ^3^BCS, Body condition scores. ^4^DMI, Dry matter intake. ^5^TAG, triacylocytes treatment.

**Supplemental Fig. S1.** Effects of NAC on the oxidation and antioxidant enzyme systems in BMECs. **(A)** Western blotting analysis of Nrf2, with β-actin used as a loading control. **(B)** Quantification of Nrf2. **(C)** Nrf2 immunostaining. **(D)** H_2_O_2_ content. **(E)** MDA content. **(F)** LDH activity. **(G)** OFR contents. **(H)** CAT activity. **(I)** SOD activity. **(J)** GPX activity. **(K)** T-AOC. **(L)** Trx content. **(M)** TrxR activity. **(N)** GST activity. **(O)** GSH/GSSG ratio. **(P)** POD activity. The treatment group data were normalised with the control group data. Results represent the mean ± SEM from three independent replicates. ^*^ *p* < 0.05, ^**^ *p* < 0.01, ^#^ *p* < 0.05 and ^##^ *p* < 0.01.


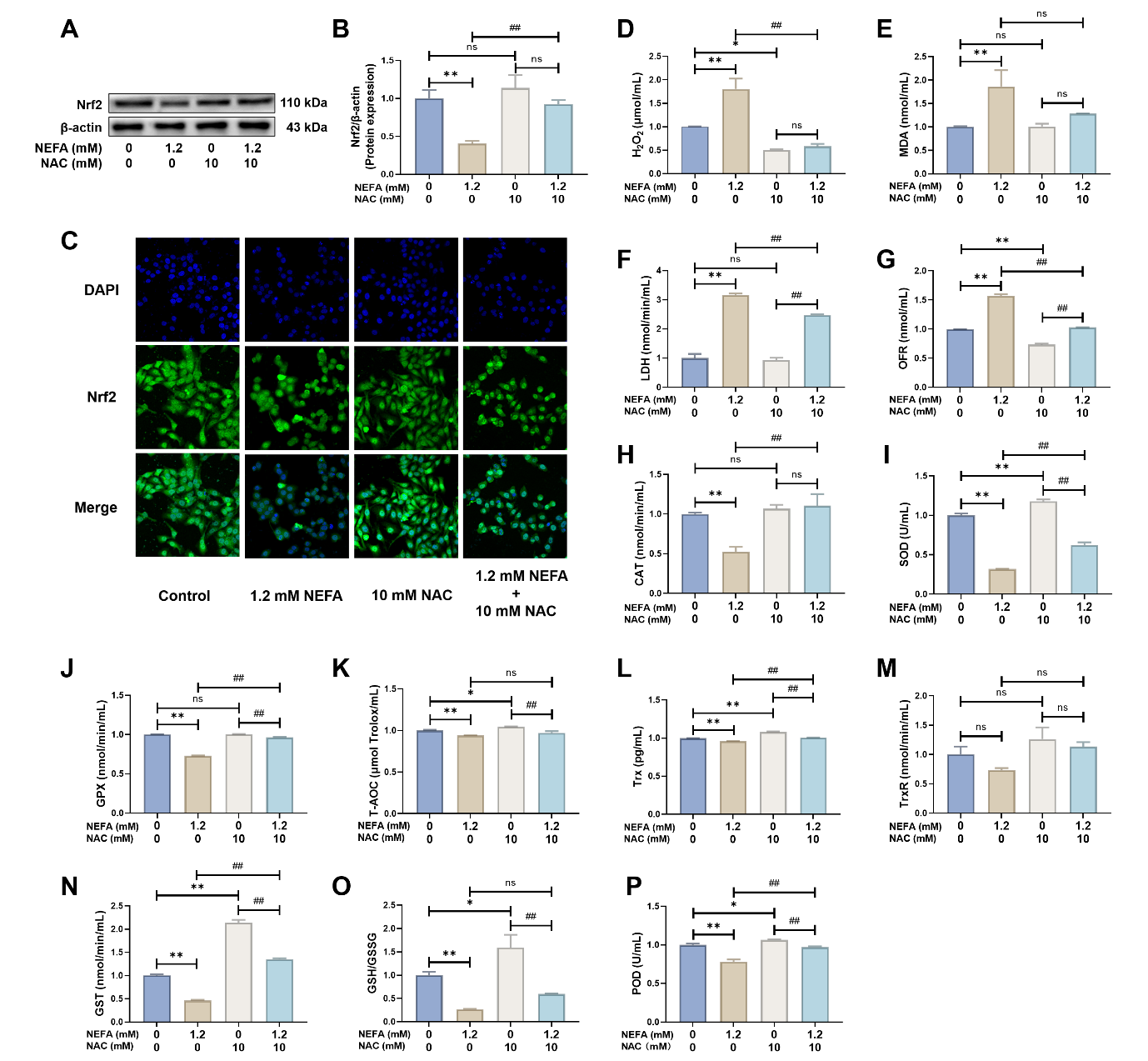


**Supplemental Fig. S2.** NAC improved the abundance of milk protein synthesis in BMECs. **(A)** Western blotting analysis of the proteins associated with milk protein synthesis, with β-actin used for normalization. **(B-F)** Quantification of the protein expressions associated with milk protein synthesis. The treatment group data were normalised with the control group data. Results represent the mean ± SEM from three independent replicates. *p* < 0.05, ^**^ *p* < 0.01, ^#^ *p* < 0.05 and ^##^ *p* < 0.01.


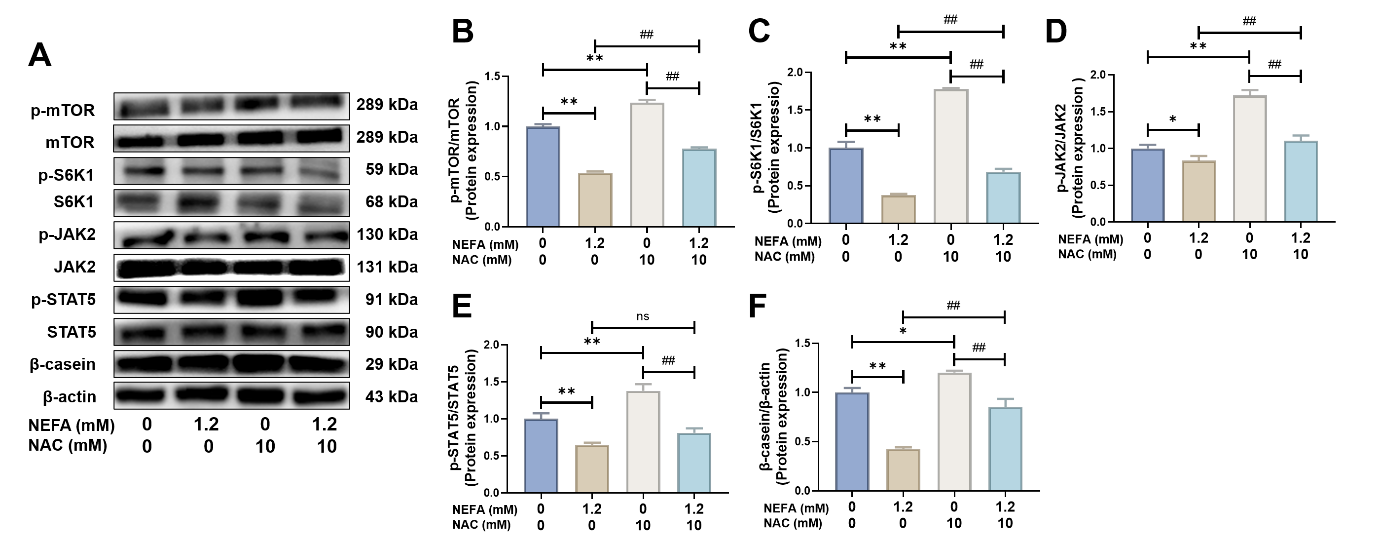

Supplement: Supplementary file 1 — Supplemental Table S1: The basal diet formulation. Supplemental Table S2: Baseline characteristics of control cows and cows with fatty liver (n = 10 per group). Supplemental Fig. S1: Effects of NAC on the oxidation and antioxidant enzyme systems in BMECs. (A) Western blotting analysis of Nrf2, with β‐actin used as a loading control. (B) Quantification of Nrf2. (C) Nrf2 immunostaining. (D) H2O2 content. (E) MDA content. (F) LDH activity. (G) OFR contents. (H) CAT activity. (I) SOD activity. (J) GPX activity. (K) T‐AOC. (L) Trx content. (M) TrxR activity. (N) GST activity. (O) GSH/GSSG ratio. (P) POD activity. The treatment group data were normalised with the control group data. Results represent the mean ± SEM from three independent replicates. *p < 0.05, ** p < 0.01, #p < 0.05 and ## p < 0.01. Supplemental Fig. S2: NAC improved the abundance of milk protein synthesis in BMECs. (A) Western blotting analysis of the proteins associated with milk protein synthesis, with β‐actin used for normalization. (B–F) Quantification of the protein expressions associated with milk protein synthesis. The treatment group data were normalised with the control group data. Results represent the mean ± SEM from three independent replicates. *p < 0.05, **p < 0.01, #p < 0.05 and ##p < 0.01. [file VMS3-12-e71065-s001.docx]
